# One-to-one versus group setting for conducting computer-assisted TTO studies: findings from pilot studies in England and the Netherlands

**DOI:** 10.1007/s10198-013-0509-9

**Published:** 2013-07-31

**Authors:** Koonal K. Shah, Andrew Lloyd, Mark Oppe, Nancy J. Devlin

**Affiliations:** 1 Office of Health Economics, 7th Floor Southside, 105 Victoria Street, London, SW1E 6QT UK; 2 School of Health and Related Research, University of Sheffield, Regent Court, Sheffield, S1 4DA UK; 3 Oxford Outcomes, Seacourt Tower, West Way, Oxford, OX2 0JJ UK; 4 Department of Health Policy and Management, Institute for Medical Technology Assessment, Erasmus University Rotterdam, Burgemeester Oudlaan 50, 3000 DR, 3062 PA Rotterdam, The Netherlands

**Keywords:** Time trade-off, Mode of administration, Preference elicitation, EQ-5D, Interviewer effect, I100

## Abstract

We compare two settings for administering time trade-off (TTO) tasks in computer-assisted interviews (one-to-one, interviewer-led versus group, self-complete) by examining the quality of the data generated in pilot studies undertaken in England and the Netherlands. The two studies used near-identical methods, except that in England, data were collected in one-to-one interviews with substantial amounts of interviewer assistance, whereas in the Netherlands, the computer aid was used as a self-completion tool in group interviews with lesser amounts of interviewer assistance. In total, 801 members of the general public (403 in England; 398 in the Netherlands) each completed five TTO valuations of EQ-5D-5L health states. Respondents in the Netherlands study showed a greater tendency to give ‘round number’ values such as 0 and 1 and to complete tasks using a minimal number of iterative steps. They also showed a greater tendency to skip the animated instructions that preceded the first task and to take into account assumptions that they were specifically asked not to take into account. When faced with a pair of health states in which one state dominated the other, respondents in the Netherlands study were more likely than those in the England study to give a higher value to the dominant health state. On the basis of these comparisons, we conclude that the one-to-one, interviewer-led setting is superior to the group, self-complete setting in terms of the quality of data generated and that the former is more suitable than the latter for TTO studies being used to value EQ-5D-5L.

## Introduction

The valuation of health-related quality of life, as required for the estimation of quality-adjusted life years, generally entails asking members of the general public to imagine how good or bad it would be to live in particular health states. Stated preference methods commonly used for this purpose, such as time trade-off (TTO) and standard gamble, ask respondents which choices they say they would make when faced with hypothetical decisions and scenarios. In TTO, for example, the respondent faces a choice between two hypothetical ‘lives’ (one involving a period of time in an impaired health state; the other involving a shorter period of time in full health), and their valuation of the impaired health state is calculated according to how much time in full health they say that they would give up in order to avoid the life involving that state.

Health state valuation exercises have conventionally been undertaken in face-to-face interviews in a one-to-one setting. For example, in the measurement and valuation of health (MVH) study conducted by Dolan et al. [1], each respondent was interviewed in their own home by a trained interviewer who used physical props (such as a specially designed double-sided TTO board) to guide the respondent through each task. The MVH approach is extremely resource intensive, and developments in computer technology have the potential to reduce the data collection burden [2, 3]. Computer-based surveys can be custom-designed to present information and collect choice data in a clear, user-friendly manner. The TTO method may be particularly well-suited to computer-assisted administration because of the capability to programme a series of ‘iterative steps’ which guide respondents towards their desired valuation (given by their point of indifference between the two hypothetical lives). Including TTO tasks as part of web-based surveys could potentially offer a cost-effective means of collecting a large amount of data in a very short period of time.

However, since TTO is a cognitively challenging task [4], it has generally been assumed that a trained interviewer must be present to provide instruction and guidance in order to ensure data quality. It is also clear that the mode of administration can have serious effects on data quality [5]. For example, in a randomised study comparing two modes of administering valuations of EQ-5D health states using TTO (face-to-face, interviewer-led vs. online, self-complete), Norman et al. reported that the online arm yielded larger proportions of central and extreme values (0, 1 and −1) than did the face-to-face arm [3]. However, while online self-completion of TTO tasks with no interviewer assistance may be problematic, alternative settings, such as a group interview session where respondents self-complete computer-based surveys with interviewers on hand to provide instruction and assistance as required, offer a potentially attractive compromise.

Over the past few years, the EuroQol Group has been preparing for the valuation of the EQ-5D-5L, an expanded level version of its standardised instrument for measuring health-related quality of life [6]. This new instrument has five levels to describe the nature of health problems on each dimension. As part of a programme of research to develop and test new methods for valuing EQ-5D-5L, the EuroQol Group commissioned studies in eight countries to pilot EQ-5D-5L valuation interviews. In these interviews, a series of discrete choice and TTO tasks were presented and completed using a fully automated computer aid, the EuroQol valuation technology (EQ-VT). EQ-VT captured and time stamped all respondent actions, meaning that, in addition to the health state values themselves, we have access to a rich underlying data set on the process that respondents followed in arriving at those values. The pilot studies in England and the Netherlands used near-identical methods, except that in England, data were collected in one-to-one interviews with substantial amounts of interviewer assistance, whereas in the Netherlands, EQ-VT was used as a self-completion tool in group interviews with lesser amounts of interviewer assistance.

The aim of this paper was to compare these two settings for administering TTO tasks in computer-assisted interviews (one-to-one, interviewer-led vs. group, self-complete) by examining the quality of the data generated in the pilot studies.

## Methods

### Study design

Two separate studies were undertaken, one in England and the other in the Netherlands. Both studies consisted of the following components (in order): self-reported health using EQ-5D-5L; self-reported health using the EuroQol visual analogue scale (EQ-VAS); basic background questions; 10 discrete choice tasks in which respondents were asked to choose which is the better of a pair of EQ-5D-5L health states, followed by rating of both of those health states using VAS; structured feedback questions regarding the discrete choice tasks; five TTO tasks; structured feedback questions regarding the TTO tasks; further background/sociodemographic questions. Both the first discrete choice task and the first TTO task were preceded by animated instruction sequences which explained how the tasks worked and what was required of the respondent. This paper focuses on the elements that are relevant to the valuation of health states using TTO.

In order to generate positive and negative values using a uniform elicitation procedure, an alternative approach to TTO, the lead-time TTO [7], was used. This approach involves adding time in full health (‘lead time’) to both of the lives being compared (Life A and Life B), allowing respondents to ‘trade into’ these additional years of full health when they consider the health state being valued to be worse than dead. Lead-time TTO has been shown to be feasible for the valuation of EQ-5D health states [8]. Each task involved a lead time of 10 years, followed by a health state duration of 5 years, giving a 15-year time frame. This variant of lead-time TTO is the same as that used in an earlier exploratory study [9]. The minimum value that can be produced directly from this variant is −2. No additional trade-off questions were asked of those who ‘exhausted’ their lead time.

**Fig. 1:**
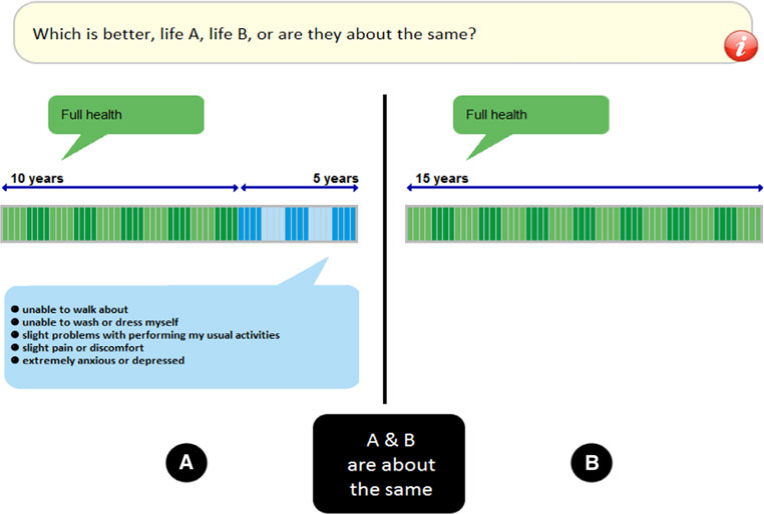
Screenshot of the lead-time time trade-off (TTO) as it appeared in EQ-VT

All TTO tasks were implemented in EQ-VT. The England study used an English language version of EQ-VT, whereas the Netherlands study used a Dutch language version. All other aspects of EQ-VT were exactly the same across the two studies. An example of the visual display of the lead-time TTO task in the English language version is shown in Fig. 1. In each task, respondents were first presented with a choice between 10 years in full health followed by 5 years in the health state to be valued (Life A) versus 15 years in full health (Life B). This choice is depicted in Fig. 1. We would expect most respondents to prefer Life B to Life A, since this implies that they prefer full health to a state of ill health. If this is the case, then clicking the ‘B’ button triggers a change in the amount of time in full health in Life B such that the choice is then between 10 years in full health followed by 5 years in the health state to be valued (Life A) versus 10 years in full health (Life B). According to the theory underpinning the lead-time TTO approach, respondents’ response to this choice indicates whether they consider the health state to be better or worse than dead [9].

Each TTO task ends only when the respondent clicks the button indicating that they consider Life A and Life B to be ‘about the same’. At this point of indifference, the implied health state value is calculated by subtracting 10 (the number of years of lead time) from the total number of years in Life B, then dividing by five (the total number of years in Life A, minus the number of years of lead time). This can be expressed as *U* = (*t* − 10)/5, where *U* is the health state value (utility) and *t* is the number of years in Life B at the respondent’s point of indifference. The maximum value of 1 (respondent’s point of indifference occurs when Life B involves 15 years in full health; *t* = 15) implies that the respondent considers the health state to be as good as being in full health. The minimum value of −2 (respondent exhausts all of their lead time—that is, they do not prefer Life A even when Life B involves zero years; *t* = 0) implies that the respondent considers the health state to be so undesirable that they would rather die immediately than live for 10 years in full health followed by five years in the health state. A value of zero (respondent’s point of indifference occurs when Life B involves 10 years of full health; *t* = 10) implies that the respondent considers the health state to be no better and no worse than dead.

**Fig. 2:**
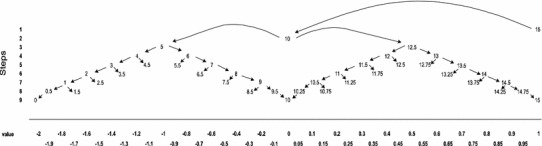
Iterative process used for arriving at point of indifference in the TTO task

The automated iterative routing used to seek the point of indifference was based on an adaptation of the MVH approach and is reproduced in Fig. 2. Respondents were able to ‘change their mind’ at any point by either reversing the direction of their trading or by clicking a ‘reset’ button which enabled them to re-start the task.

A blocked design was used to select 100 health states (from the 3,125 defined by the EQ-5D-5L) for valuation via TTO and to allocate respondents to blocks which each comprised a set of five health states. Each respondent received a combination of mild, moderate and severe health states.

### Sample and mode of administration

In each study, approximately 400 members of the general public were recruited to be broadly representative of the general populations of the relevant countries in terms of age and gender. The samples were recruited by approaching members of a panel of individuals (belonging to the agencies responsible for fieldwork in each country) who had previously indicated a willingness to participate in research studies.

In the England study, a cash payment of £15 was offered as an incentive for participation. Face-to-face interviews were undertaken by a team of eight interviewers who completed one-to-one interviews in the homes of the respondents in different areas of the country. Although the respondent was in control of the computer (laptop) throughout the tasks, the interviewer guided them through each step. The one-to-one setting allowed interviewers to provide detailed instruction and feedback to the respondent where appropriate.

In the Netherlands study, a cash payment of €27.50 was offered (€20 for participation plus €7.50 to cover travel costs). Groups of between 15 and 25 respondents attended each interview session, all of which took place in the city of Amersfoort. At the start of the session, the respondents were given an introduction and explanation of the tasks (by the lead researcher) as a group; they were then asked to self-complete the tasks at individual computer terminals, with assistance provided by three interviewers (the lead researcher and two assistants) when required. In both studies, the interviewers were very experienced in conducting TTO interviews from previous studies and had completed training on the specifics of the methodology and procedures for this particular study.

### Methods of analyses

We identified a priori a number of indicators and analyses that could be used to compare the quality of the data generated in the two studies. Completion rates were compared, although we were unable to capture the specific reason(s) for non-completion. We compared the overall distributions of TTO valuations, identifying in particular the proportions of respondents giving potentially problematic valuations such as 1 (implies that the respondent considers the health state to be equivalent to being in full health; often referred to as ‘non-trading’), 0 (implies that the respondent considers the health state to be equivalent to dead) and −2 (the respondent exhausts all of the lead time available to them).

From the blocked design, we identified pairs of health states faced by the same respondent whereby one of the states could be considered to dominate the other. We then compared the number of times that respondents gave valuations that were inconsistent with the logical ordering of the relevant health states.

In order to assess respondents’ levels of engagement and tendency to take ‘shortcuts’ during the TTO exercise, we compared the number of steps and the amount of time taken to complete each task and examined the extent to which respondents engaged in the choice iteration process in order to arrive at their point of indifference. Finally, we compared respondents’ stated levels of understanding by examining their responses to the structured feedback questions. Differences between studies were evaluated using chi-squared tests of associations at the 5, 1 and 0.1 % levels of significance.

## Results

A total of 819 respondents were recruited to the study (England *n* = 407; Netherlands *n* = 412). Of these, 18 respondents (4 from the England study and 14 from the Netherlands study) did not complete all of the valuation tasks and were excluded from the final analysis. There is statistically significant evidence of an association between setting and the propensity to complete all of the valuation tasks (*p* < 0.05). Complete valuation data are therefore available for 801 respondents (England *n* = 403; Netherlands *n* = 398). A number of respondents are shown as having data for more than five TTO tasks (37 respondents in England; 117 respondents in the Netherlands). This is due to the functionality in EQ-VT to re-start any given task. For the purposes of this paper, only the final valuation for each task is included in the data analysis. However, it is noteworthy that the tendency to re-start TTO tasks was much greater in the Netherlands study.

The Netherlands sample was slightly younger than the England sample overall (Table 1). The two samples were similar in terms of gender and whether respondents had experience of serious illness.

**Table 1:** Background characteristics of the samples used

Variable	England	Netherlands	Overall
Number of respondents (only those who completed interviews)	403	398	801
Mean age (years)	42.0	36.3	39.1
Gender (%)				
Male	48.5	50.1	49.3
Female	51.5	49.9	50.7
Question: ‘Do you have experience of serious illness... (%)				
… in you yourself	15.6	18.4	17.0
… in your family	68.8	67.5	68.2
… in caring for others	35.2	28.8	32.0

The majority of interviews in the England study (96.5 %) were conducted by eight interviewers. All interviews were completed in a one-to-one setting. In the Netherlands study, all interviews were completed in three group sessions. There was considerable variation in sample composition across interviewers. For example, in one of the Netherlands group sessions only a third of respondents were male, compared to nearly 70 % in another of the sessions. The mean duration of the valuation task components of the interview (discrete choice tasks + TTO tasks combined) was 20 min overall. This varied across the interviewers, with the average duration per interviewer ranging from 16 to 27 min.

### Distribution of TTO valuations

**Fig. 3:**
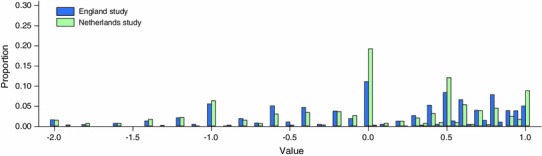
Valuation distribution across all TTO tasks and health states

Figure 3 shows the overall distribution of TTO valuations for all five tasks combined. In both studies, there was a clustering of valuations around whole numbers of years. For example, a large proportion of respondents chose points of indifference when Life B was equal to 5 years, translating to a health state value of −1. However, very few respondents chose points of indifference when Life B was equal to either 4.5 or 5.5 years, which translate to values of −1.1 and −0.9, respectively.

Across all health states, the modal point of indifference was when Life B was equal to 10 years, translating to a value of 0. As noted above, this implies that the respondent considered the health state to be equivalent to dead. The tendency towards this indifference point was stronger in the Netherlands study, where nearly 20 % of all health states were valued in this way. In the Netherlands study, 48.2 % of respondents gave a zero valuation in at least one of their five TTO tasks, compared to 37.5 % of respondents in the England study. There is statistically significant evidence of an association between setting and the propensity to give a zero valuation to a given health state (*p* < 0.001).

This proportion of health states that were valued as worse than dead did not differ greatly across the two studies (33.4 % in the England study; 30.2 % in the Netherlands study), although there is some evidence of an association between setting and the propensity to value a given health state as being worse than dead (*p* < 0.05). Respondents chose points of indifference when Life B was zero years (implying that 10 years in full health followed by 5 years in the health state is no better than immediate death) on 65 occasions (1.6 % of all valuations). While this tendency to exhaust lead time was similar across both studies (*p* > 0.05), ‘non-trading’ was more common in the Netherlands study—8.8 % of all health states were valued as being equivalent to full health (value of 1) compared to 5.1 % in the England study (*p* < 0.01).

### Logical consistency

As a result of the blocked design, the majority of respondents were presented with at least one pair of TTO health states whereby one of the states could be considered to dominate the other. For example, respondents who valued health state 11445 also valued health state 25455. The latter is worse than the former on three dimensions and no better on the other two dimensions, and so is overall a logically worse state. These pairs of dominant/dominated health states allowed us to test whether respondents’ TTO valuations were consistent with the logical ordering of the relevant health states. Overall, 9.4 % of valuations of dominant/dominated pairs failed this test of logical consistency. The proportion of inconsistent valuations was larger in the Netherlands study (10.7 %) than in the England study (8.3 %), although the association between setting and the propensity to give a pair of logically inconsistent values is not statistically significant at the 5 % level.

However, it should be noted that only 24 respondents (less than 4 % of the total sample; 9 in the England study, 15 in the Netherlands study) gave such inconsistent valuations, with some of these respondents giving inconsistent valuations for multiple pairs of health states. This occurred when respondents were included in a block which contained two or more dominant/dominated pairs.

### Valuation process

The mean number of iterative steps taken to complete a TTO task was 6.65. While this statistic was similar in both studies, respondents in the Netherlands study were more likely than those in the England study to use either very few or very many steps, as shown in Table 2. There is statistically significant evidence of an association between setting and the propensity to complete tasks using five steps or fewer (*p* < 0.01). The range was very high, with a small minority of respondents in both studies using ≥50 steps to achieve indifference.

**Table 2:** Number of steps taken to complete time trade-off tasks

Number of steps taken to complete task	Frequency,* n* (%)	
	England	Netherlands
1–5 steps	857 (43)	1,040 (52)
6–10 steps	962 (48)	691 (35)
11–20 steps	171 (8)	204 (10)
≥21 steps	25 (1)	55 (3)
Mean number of steps taken	6.73	6.57
Median number of steps taken	6.00	6.00

Table 3 summarises the amount of time taken to complete TTO tasks. The data are split according to task order, with one column for the first task and another for the second and subsequent tasks combined. This is because the time taken to complete the first task includes the animated instruction sequence as well as the time taken to actually value the health state.

**Table 3:** Amount of time taken to complete time trade-off tasks

Amount of time taken to complete task (sec)	Frequency,* n* (%)			
	England		Netherlands	
	First task	Second and subsequent tasks	First task	Second and subsequent tasks
0–30	4 (1)	440 (27)	23 (6)	730 (46)
30–60	16 (4)	708 (44)	41 (10)	642 (40)
60–120	27 (7)	383 (24)	82 (21)	194 (12)
120–240	175 (43)	73 (5)	172 (43)	24 (2)
>240	181 (45)	8 (0)	80 (20)	2 (0)
Mean number of seconds taken	238	53	168	41
Median number of seconds taken	228	40	169	30

Overall, respondents in the Netherlands study took less time to complete TTO tasks than did those in the England study, with nearly half of the second and subsequent tasks completed within 30 s. The mean amount of time taken for respondents in the Netherlands study to complete the first task (168 s) is considerably lower than the equivalent statistic for those in the England study (238 s). This may be linked to the fact that respondents in the Netherlands study were able to skip some or all of the animated instruction sequence. It is reasonable to assume that respondents would be more likely to skip the instructions in the group, self-complete setting than in the one-to-one setting with greater interviewer supervision. When viewed in its entirety, the animated instruction sequence lasted for approximately 135 s. Yet 146 respondents in the Netherlands study (36.7 %) completed the first task in less than 120 s (compared to 12 % of respondents in the England study), indicating that they skipped at least part of the instructions. There is statistically significant evidence of an association between setting and the propensity to complete the first task in less than 120 s (*p* < 0.01).

By examining the process by which respondents reached their TTO indifference point, we were able to categorise each valuation task into various ‘iterative types’ (Table 4).

**Table 4:** Summary of all time trade-off valuation tasks, by ‘iterative type’

Iterative type	Frequency,* n* (%)	
	England	Netherlands
No iteration	33 (1.6)	135 (6.7)
Iteration in a single direction	337 (16.6)	502 (25.0)
Iteration with only one reversal	937 (46.3)	827 (41.2)
Iteration with multiple reversals	718 (35.5)	545 (27.1)

Table 4 contains two striking observations. First, failing to iterate was over fourfold more common in the Netherlands study than in the England study. This difference supports the previous finding that non-trading occurred more frequently in the Netherlands study, but it should be added that respondents in the England study were more likely to undertake some iteration before eventually returning to the start point (yielding a value of 1) and choosing this as their indifference point. Second, respondents in the Netherlands study were far more likely to complete tasks with iteration in a single direction, which indicates that they did not prefer Life A at any stage in the task. It should also be noted that 38 respondents in the Netherlands study attempted to choose Life A in the first step (implying that they preferred 10 years in full health followed by 5 years in the impaired health state to 15 years in full health—the EQ-VT did not permit this choice). There were no observations of respondents in the England study choosing in this way.

### Structured feedback questions

After completing all five TTO tasks, respondents were asked to answer a set of feedback questions in which they used a five-level Likert item to specify their level of agreement (1 = agree; 5 = disagree) with statements about their experience of the TTO exercise. Table 5 summarises the responses to these feedback questions.

**Table 5:** Tabulated summary of responses to structured feedback questions relating to time trade-off

Statement	% Of respondents who gave responses of 1 or 2 (i.e. who agreed with the statement)	
	England	Netherlands
1. The instructions that were given on the computer made it clear what I needed to do	96	88
2. It was easy to understand the questions I was asked	95	85
3. I found it difficult to decide on the exact point where Life A and Life B were about the same	70	64
4. I found it easy to tell the difference between the health states I was asked to think about	75	66
5. The number of years in these ‘lives’ was too long to be meaningful to me	10	20
6. When I was asked to think about living in very poor health, I imagined that some treatment or relief would be provided	26	40
7. When you live in very poor health for a long time, you can get used to it and learn to live with the health problems	55	67
8. When I thought about poor health, whether I would still be able to work was an important consideration for me	34	36
9. I found it difficult to imagine what it would be like to live these ‘lives’	50	41

While the majority of respondents in both studies indicated that they found the TTO tasks and instructions clear and easy to understand, respondents in the England study were more likely than those in the Netherlands study to do so. There is statistically significant evidence of an association between setting and the propensity to agree with the first and second statements in Table 5 (*p* < 0.01 and *p* < 0.01, respectively). By contrast, respondents in the Netherlands study were much more likely than those in the England study to indicate that they took into account the possibility that some treatment or relief would be provided (there is statistically significant evidence of an association between setting and the propensity to agree with the sixth statement in Table 5; *p* < 0.01). This is in spite of the fact that the pre-task instructions stated that “you must not imagine that the condition will improve in any way, for example, by taking painkillers, or because of new medical discoveries or by dying sooner than 5 years”.

## Discussion

In this paper, we have compared the results of two pilot studies (undertaken in England and the Netherlands) in which a series of TTO tasks was administered in computer-assisted interviews. The studies followed similar protocols and used the same computer aid, but differed in that in England, interviews were undertaken in the one-to-one setting with substantial amounts of interviewer assistance, whereas in the Netherlands, they were undertaken in the group, self-complete setting with lesser amounts of interviewer assistance. Comparing the results of these studies has allowed us to examine the effect that the interview setting had on data quality and on perceived levels of respondent effort and understanding.

At the outset of the project, we identified a number of ways in which to compare the results of the two studies. Based on the data supporting these comparisons, it is clear that the one-to-one, interviewer-led setting (as used in the England study) is superior to the group, self-complete setting. The Netherlands study was associated with higher levels of respondent drop-out (defined in terms of failure to complete all of the valuation tasks). Respondents in the Netherlands study showed a greater tendency to complete tasks with minimal iteration (that is, using a very small number of iterative steps), thereby failing to use much of the Life B scale that was available to them. This resulted in large numbers of observations at ‘round number’ values such as zero (indicating that the health state is no better and no worse than dead), 0.5 and 1 (non-trading). This is consistent with findings elsewhere in the literature [3] which suggest that the tendency to give such central and/or extreme values is influenced by the mode of administration.

When faced with a pair of health states in which one state dominated the other, respondents in the Netherlands study were more likely than those in the England study to give a higher value to the dominant health state. They were also more likely to skip the animated instructions that preceded the first TTO task. This may in turn be linked to their responses to the follow-up feedback questions, which suggest that these respondents were more likely to take into account an assumption that they were specifically asked not to take into account during the instructions. The proportion of respondents indicating that they took into account the possibility of treatment or relief (despite being instructed not to do so) was lower than that observed in a previous study where the same structured feedback question was asked [9], although this may be due to the fact that in the present study respondents were presented with fewer severe health states. Nevertheless, it remains worrying that some respondents continue to take this into account when completing TTO tasks, so further work needs to be done in terms of strengthening instructions, particularly if a group interview setting is to be used.

The evidence presented in this paper highlights the importance of the role of the interviewer in TTO studies in terms of providing instruction and guidance to the respondent. This is consistent with the findings of Edelaar-Peeters et al. [10], who examined the importance of interviewer assistance in a web-based TTO study in which respondents were asked to value six EQ-5D health states. The authors found that 86 % of the sample sought help from an interviewer at least once, with the most common reasons for requiring assistance reflecting a failure to understand the TTO correctly. It is reasonable to assume that such misunderstandings would be easier to identify and resolve in a one-to-one, interviewer-led setting than in a group, self-complete setting.

However, there are a number of caveats. First, interviews undertaken in the one-to-one setting are more resource intensive than those undertaken in the group setting. By definition, the one-to-one setting requires only one trained interviewer to conduct each individual interview, whereas in the group setting, one interviewer can conduct multiple interviews concurrently. Second, the use of interviewers can lead to forms of bias–for example, the interviewer may give subtle clues that influence the respondent towards certain preferences or choice strategies. Such bias is likely to be more problematic in the one-to-one setting where there is expected to be greater interaction between interviewers and respondents. Third, while TTO is acknowledged to be a cognitively challenging exercise, other approaches to valuing health states may be more conducive to completion in a group setting (VAS, for example, has been described as the most feasible and acceptable of the health state valuation methods [11]).

There may also be differences between the studies, other than whether the interviews were undertaken in the one-to-one or group setting, that could have driven differences in the results. These include unobserved differences between the samples (in terms of either the extent to which the samples were truly representative of the general populations from which they were recruited, or the extent to which there exist inherent differences between people living in England and people living in the Netherlands) and between the interviewers (for example, differences in levels of experience or enthusiasm).

Language is another consideration. In the Netherlands study, all aspects of the interview were carried out in Dutch. The EuroQol Group operates a rigorous translation process according to a standard protocol that conforms to internationally recognised guidelines. The guidelines aim to ensure equivalence to the English source version and involve a forward/backward translation process and cognitive debriefing [12, 13]. Nevertheless, it is inevitable that there will be some elements of inconsistency across translations. For example, the fourth and fifth levels of the pain/discomfort dimension in the English version of EQ-5D-5L refer to ‘severe’ and ‘extreme’ problems, respectively. In the Dutch version of EQ-5D-5L, these levels refer to ‘ernstig’ and ‘extreem’ problems. Unless the Dutch terms can be considered to be exactly equivalent to their English counterparts, it is possible that the observed valuations may have been influenced by the ways in which the terms were interpreted by respondents.

Finally, it should be noted that some of our concerns about the Netherlands study data (such as the tendency towards round number valuations) also apply to the England study data, albeit to a lesser degree. These may be linked to other aspects of the methodology such as the use of EQ-VT (for example, did the computer aid make round number valuations more attractive, in terms of visual prominence or the ease of arriving at those valuations?). The large number of zero valuations (observed in both the England study and the Netherlands study), for example, has not always been apparent in other TTO studies (although it should be noted that the valuation distribution observed in the MVH study was also characterised by a peaks at −1, 0 and 1 [8]). Hence, the data quality issues cannot be attributed solely to the choice of setting.

## Conclusion

The majority of the evidence presented in this paper suggests that the one-to-one, interviewer-led setting (as used in the England study) generates higher quality data than the group, self-complete setting (as used in the Netherlands study). We therefore conclude that the former setting is more suitable than the latter setting for valuing EQ-5D-5L and that unsupervised settings such as web-based surveys are unlikely to be suitable for TTO or other complex valuation techniques.
